# Prevalence and Drivers of COVID-19 Vaccine Booster Hesitancy Among German University Students and Employees

**DOI:** 10.3389/fpubh.2022.846861

**Published:** 2022-04-07

**Authors:** Sameh Attia, Katharina Mausbach, Miloslav Klugar, Hans-Peter Howaldt, Abanoub Riad

**Affiliations:** ^1^Department of Oral and Maxillofacial Surgery, Faculty of Medicine, Justus-Liebig-University, Giessen, Germany; ^2^Department of Prosthodontics, Faculty of Medicine, Justus-Liebig-University, Giessen, Germany; ^3^Czech National Centre for Evidence-Based Healthcare and Knowledge Translation (Cochrane Czech Republic, Czech EBHC: JBI Centre of Excellence, Masaryk University GRADE Centre), Faculty of Medicine, Institute of Biostatistics and Analyses, Masaryk University, Brno, Czechia; ^4^Department of Public Health, Faculty of Medicine, Masaryk University, Brno, Czechia

**Keywords:** cross-sectional studies, COVID-19 vaccines, decision making, Germany, social determinants of health, vaccination hesitancy

## Abstract

COVID-19 booster hesitancy (VBH) is a serious public health challenge which acts simultaneously with the waning vaccine-elicited immunity and the emerging viral variants to prolong the pandemic interval. Therefore, this study aimed to evaluate the prevalence of COVID-19 VBH among a highly educated subset of the German population and to explore the potential demographic, anamnestic, and psychosocial determinants of this problem. A cross-sectional survey-based study was conducted in December 2021 among German university students and employees to evaluate their attitudes toward COVID-19 vaccine booster (VB) doses. The study used a self-administered questionnaire that was developed and disseminated digitally, and the questionnaire inquired about participants' demographic characteristics, COVID-19-related anamnesis, COVID-19 vaccine-related anamnesis, and psychosocial predictors of COVID-19 VBH. A total of 930 participants were recruited, of which 608 (65.4%) were students, 682 (73.3%) were females, and their mean age was 29.08 ± 10.93 years. Fifty-five participants (5.9%) had been previously infected by COVID-19 and the vast majority of infections happened before the first vaccine dose. Over 95% of the participants had received at least one vaccine dose, and the most commonly administered vaccine was BNT162b2. The overall COVID-19 VB acceptance was satisfactory (87.8%) and induced by various altruistic promoters, e.g., family health protection, community health protection, and patients' health protection. The students (86.3%), the previously infected participants (76.4%), the participants who did not receive primer doses of COVID-19 vaccines (2.5 %), and those who were hospitalized (40%) and sought medical care/treatment after receiving primer doses (86.8%) were less likely to accept COVID-19 VB compared to the employees (90.7%), the participants who were not previously infected (88.6%) and those who received primer dose (91.7%), and the participants who were not hospitalized (92%) nor sought medical care/treatment after primer doses (92.9%), respectively. The perceived effectiveness of COVID-19 VB against severe illness (adjusted odds ratio “AOR”: 47.65–95% confidence interval “CI”: 23.65–96.49), symptomatic infection (AOR: 9.87–95% CI: 5.20–18.71), community transmission (AOR: 5.34–95% CI: 3.00–9.49) and emerging variants (AOR: 19.12–95% CI: 10.57–34.55) were key predictors for COVID-19 VB acceptance; therefore, it needs to be highlighted in vaccine messaging. In addition, the perceived safety of COVID-19 VB and ethical dilemmas of vaccine justice need to be addressed publicly.

## Introduction

Since the emergence of severe acute respiratory syndrome coronavirus−2 (SARS-CoV-2) and its subsequent coronavirus disease (COVID-19), it had been clear that we can only overcome such a pandemic through individual acquired immunity (1). This can be reached either by previous infection or immunization through vaccines (1). Uncontrolled spread of the disease to achieve indirect immunity (herd immunity) is associated with high morbidity and mortality rates and overwhelmed healthcare systems and personnel (1, 2). Therefore, reaching herd immunity through mass vaccination is the only appealing and effective intervention amid this pandemic (2–4). Since the authorization of first COVID-19 vaccine (BNT162b2) by the European Medicines Agency (EMA) in December 2020, a cascade of milestones in the road toward herd immunity had been achieved by the European Union (EU) member states including the Federal Republic of Germany (Germany) (5). Nevertheless, after almost 1 year of mass vaccination, the pandemic remains on march and there is no predictive data about how long we still need to overcome this catastrophe.

Several variants of concern (VoC) had been identified throughout the previous months, and they were associated with breakthrough infections and novel epidemic waves (6, 7). Omicron (B.1.1.529) is the most recent VoC reported to and declared by the World Health Organization (WHO) on November 26^th^, 2021 (7, 8). Phylogenetic studies showed that Omicron arose independently of the currently dominant Delta variant (B.1.617.2), and it has an unprecedently high number of amino acid changes in the spike protein compared to the original SARS-CoV-2 detected in Wuhan (9). Few of the 30 changes brought by the Omicron variant have phenotypic impact, e.g., increased transmissibility and immune evasion, while many changes have unclear significance (9, 10). Omicron has been spreading rapidly and detected in several countries globally including Germany, where Robert Koch Institute (RKI; Berlin, Germany) reported a total of 16,748 Omicron cases by December 30^th^, 2021 with +3,619 new cases (28%) reported since the previous day (11). Therefore, RKI recommended that the persons who have not received their primer doses of COVID-19 vaccines should be vaccinated immediately and VB doses should be available to all age groups unrestrictedly (12).

Vaccine hesitancy (VH) is defined by the WHO as “delay in acceptance or refusal of vaccines despite availability of vaccine services” and depicted as one of the top 10 threats for global health since 2019 (13, 14). In Germany, Holzmann-Littig et al. (2) found that 8.3% of German healthcare workers were hesitant to accept COVID-19 vaccination in February 2021 (2). The young age was associated with lower COVID-19 vaccine acceptance, and the acceptance levels were not different across genders (2). Vaccine booster hesitancy (VBH), waning of vaccine-elicited immunity, and emerging of SARS-CoV-2 mutations act simultaneously to prolong the pandemic interval (15, 16). Therefore, tackling vaccine hesitancy through evidence-based interventions and rapid development of COVID-19 vaccine boosters (VB) that are capable of controlling the emerging mutations are the most urgent interventions which should be approached by health systems globally nowadays (15, 17–19).

This study aimed to evaluate COVID-19 VBH among a highly educated subset of the German population, i.e., university students and employees, and to explore the potential drivers of COVID-19 VBH. The primary objective was to measure the prevalence of COVID-19 VBH among German university students and employees. The secondary objectives were: (i) to evaluate the associations between COVID-19 VB acceptance and demographic and anamnestic characteristics of the target population, (ii) to evaluate the association between COVID-19 VB acceptance and its psychosocial predictors, e.g., perceived effectiveness, perceived safety, risk-benefit ratio, and ethical barriers.

## Materials and Methods

### Design

This study had been designed as an analytical cross-sectional survey-based study using a self-administered questionnaire (SAQ) and carried out between December 7^th^ and 19^th^, 2021. The SAQ was developed and circulated digitally using KoBoToolbox (Harvard Humanitarian Initiative, Cambridge, MA, USA) to collect data from the target population about their attitudes toward COVID-19 vaccine booster doses (VB) (20). The study had been entirely conducted and reported according to the STrengthening the Reporting of OBservational studies in Epidemiology (STROBE) guidelines for cross-sectional studies (21).

### Participants

A non-random sampling technique was used as the target population was university employees and students in Germany. The participating students and employees were approached through multiple digital channels which were used for promoting the survey: (a) a mass email was centrally sent to all students and staff of Justus Liebig University Giessen (Giessen, Germany) on December 7^th^, 2021, (b) a blogpost was published on the official website of the University of Duisburg-Essen (Essen, Germany) on December 13^th^, 2021, and (c) various posts on social media platforms with a high postulated engagement of students, e.g., Twitter and Facebook were published during the recruitment interval (22).

**Figure 1:**
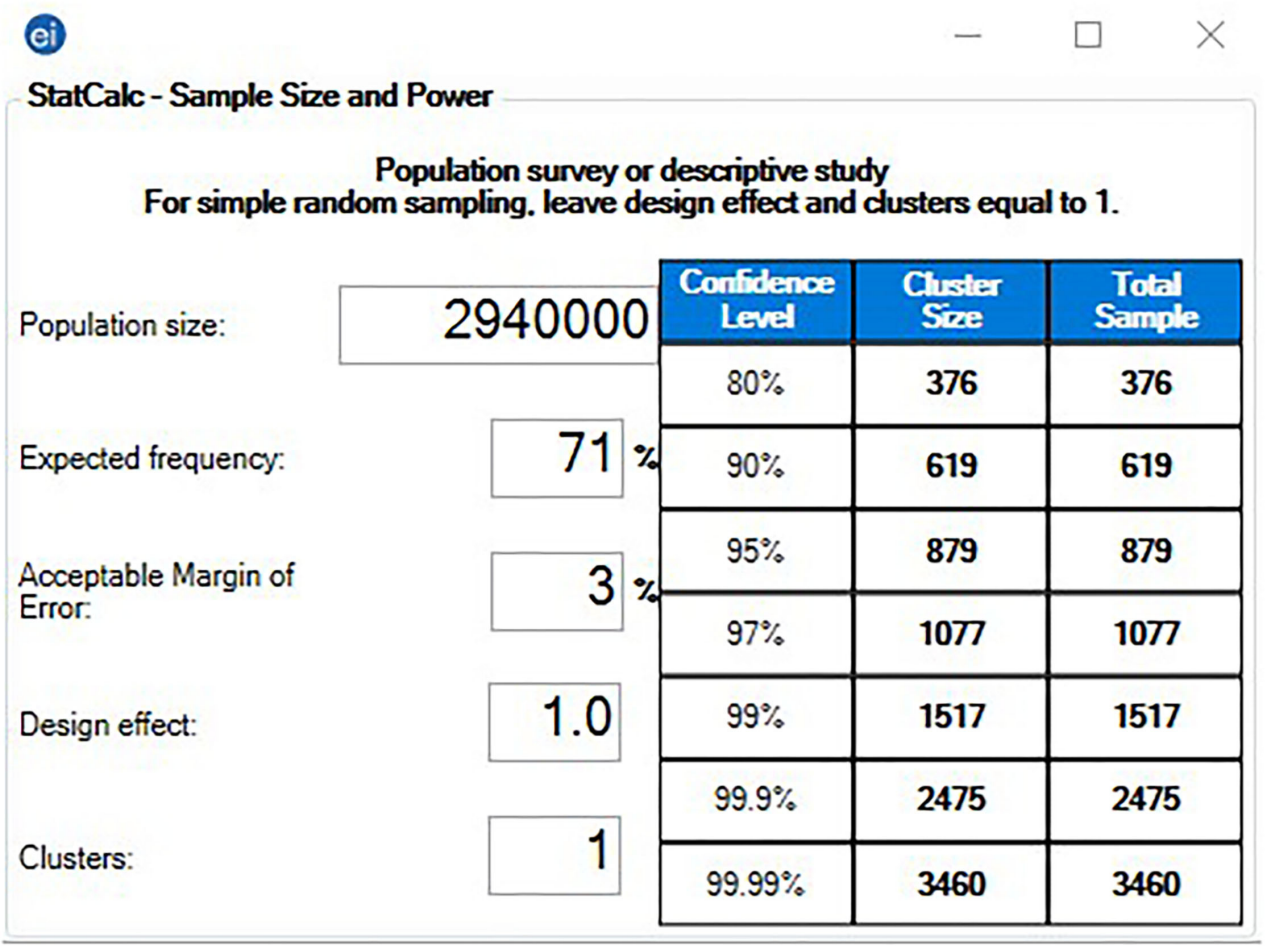
Sample size of german university students and employees—Epi-Info ^TM^ version 7.2.5.

The eligibility criteria included: (a) students who were enrolled in degree programs, and (b) academic and non-academic employees of German universities. The participants received no financial rewards or any other forms of incentives for taking part in this study. The pragmatic sample size was computed using Epi Info ^TM^ version 7.2.5 (CDC, Atlanta, GA, 2021) through the Population Survey interface (23, 24). Following the assumptions of error margin 3%, expected frequency 71% based on recent evidence of COVID-19 VB acceptance prevalence, total target population 2.94 M, and non-response rate 5%, the required sample size was 923 participants (15, 25) (Figure 1).

**Figure 2:**
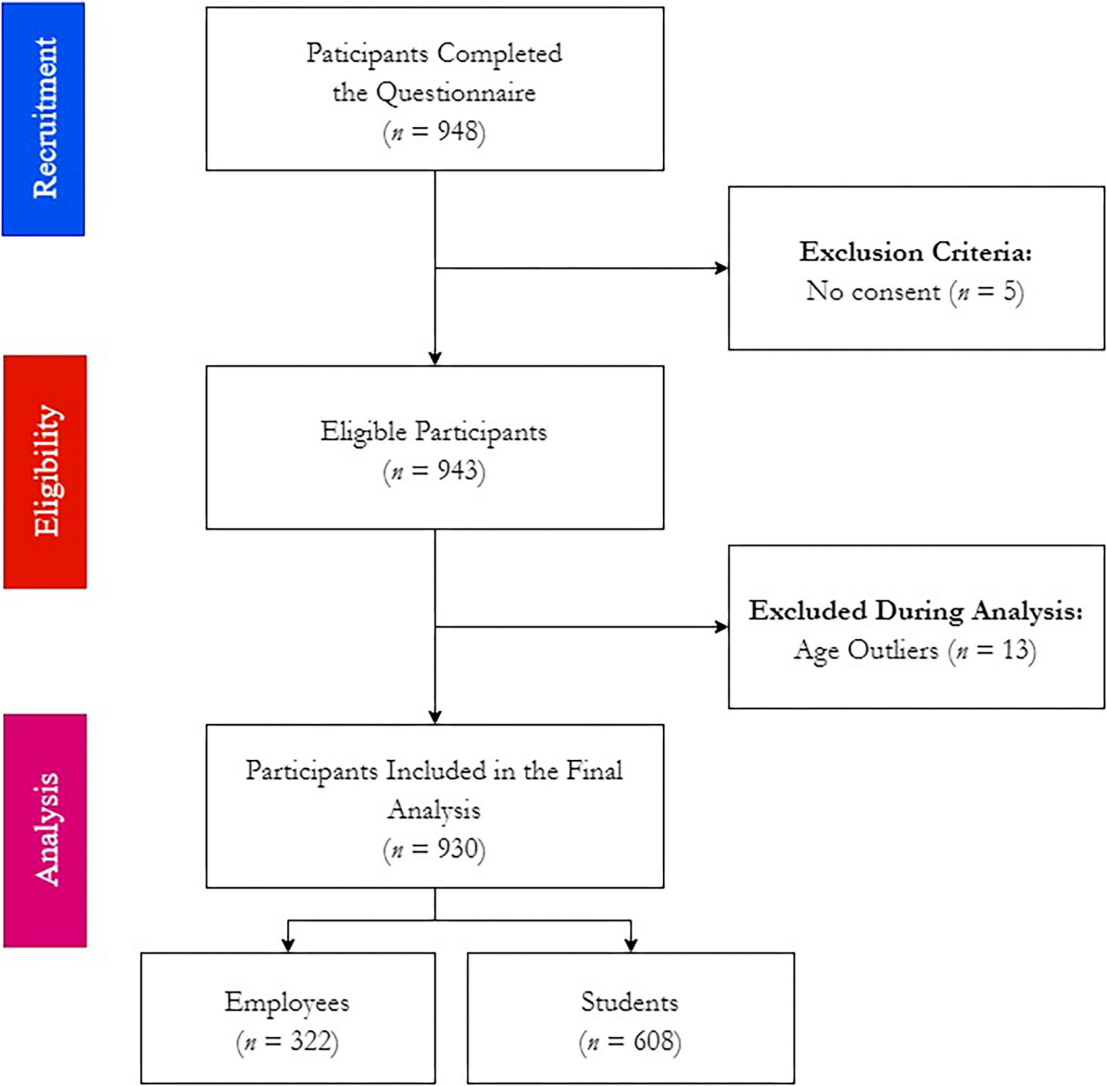
Workflow of COVID-19 Vaccine Booster Hesitancy (VBH) Survey among German university students and employees, december 2021 (*n* = 930).

By December 19^th^, 2021, 948 forms were received, of which 5 were empty responses and 13 were excluded due to being age outliers. Therefore, 930 participants were included in the downstream analyses, of which 322 were employees and 608 were students (Figure 2).

### Instrument

The used SAQ was adopted from a recent study on COVID-19 VBH among Czech healthcare workers and it exhibited a substantial level of test-re-test reliability (Cohen's κ = 0.80 ± 0.19); therefore, it was directly adopted in the current study (15, 26). A pragmatic approach for translation and cross-cultural adaptation was followed, as two German translators translated the SAQ from English to German independently, then a panel of experts evaluated and compared the two German versions in order to produce a common final German version which was used to collect data from the participants (27).

The SAQ had 19 multiple-choice items which were categorized in five sections: (i) demographic characteristics, (ii) COVID-19-related anamnesis including history of infection, onset and clinical severity, (iii) COVID-19 vaccine-related anamnesis including history of vaccination, number of doses, type of vaccine, and post-vaccination side effects, (iv) intentions to receive COVID-19 VB which were assessed by a five-point Likert scale ranging from “Totally Disagree = 1” to “Totally Agree = 5,” and (v) psychosocial drivers of COVID-19 VB acceptance, i.e., perceived effectiveness, perceived safety, perceived susceptibility and risk-benefit ratio, moral dilemma of vaccine justice, and vaccine primer dose satisfaction and vaccine selectivity (15).

### Ethics

The study protocol had been reviewed and exempted from approval by the Ethics Committee of the Faculty of Medicine at Justus Liebig University Giessen (Ref. 259/21). All participants had to provide their informed consent digitally before responding to the SAQ and they were offered to leave the study any time before finalizing the survey. The identity of the participants was kept anonymous. The Declaration of Helsinki and the General Data Protection Regulation (GDPR) had been followed during collecting, storing and handling this study data (28, 29).

### Analyses

All statistical tests were performed using the Statistical Package for the Social Sciences (SPSS) version 28.0 (SPSS Inc. Chicago, IL, USA, 2021) (30). Initially, the normal distribution of quantitative variables, e.g., age was tested using Shapiro-Wilk test with a significance level (*Sig*.) of ≤ 0.05. Then, descriptive statistics were carried out to summarize the qualitative variables using frequencies (*n*) and percentages (%), and the quantitative variables using mean and standard deviation (μ ± SD), and inter-quartile range (IQR). Consequently, inferential statistics through Chi-squared test (χ^2^), Fisher's exact test, Mann-Whitney test (*U*), and logistic regression were performed in order to test the associations between dependent and independent variables. All participants were categorized into three groups; (i) the “vaccine-acceptance” group included those who selected “Agree” or “Totally Agree” in the Likert scale question, (ii) the “vaccine hesitancy” group included those who selected “Not Sure,” and iii) the “vaccine rejection” group included those who selected “Disagree” or “Totally Disagree.” Eventually, logistic regression analysis was used to estimate the adjusted odds ratio (AOR) of psychosocial predictors for COVID-19 VB acceptance. All inferential tests were performed following the assumptions: confidence level (CI) 95%, and significance level (*Sig*.) ≤ 0.05.

## Results

### Demographic Characteristics

A total of 930 participants were included in this study, of which 682 (73.3%) were females, 232 (24.9%) were males, and 16 (1.7%) were LGBTQ+. Pregnancy was reported by only 1.5% of the participating females, and the majority of pregnant participants (70%) were in the second trimester (14–28 weeks). The mean age of the participants was 29.08 ± 10.93 (IQR: 22–31) years old which was significantly different (*Sig*. < 0.001) between the employees 38.49 ± 13.22 (IQR: 28–49) and the students 24.11 ± 4.32 (IQR: 21–26). The included participants were from 10 German states; the most represented state was Hessen (94.3%), and the least represented states were Berlin (0.1%) and Saarland (0.1%) (Table 1).

**Table 1:** Demographic characteristics of German university students and employees participating in the COVID-19 Vaccine Booster Hesitancy (VBH) Survey, december 2021 (*n* = 930).

**Variable**	**Outcome**	**Employees** **(*n* = 322)**	**Students** **(*n* = 608)**	**Total** **(*n* = 930)**	***Sig*.**
Gender	Female^†^	218 (67.7%)	464 (76.3%)	682 (73.3%)	*Reference*
	Male	100 (31.1%)	132 (21.7%)	232 (24.9%)	**0.002**
	LGBTQ+	4 (1.2%)	12 (2%)	16 (1.7%)	0.556
^†^Pregnancy	No	215 (98.6%)	457 (98.5%)	672 (98.5%)	1.000^*^
	Yes^‡^	3 (1.4%)	7 (1.5%)	10 (1.5%)	
^‡^Trimester	First trimester	1 (33.3%)	0 (0%)	1 (10%)	*Reference*
	Second trimester	2 (66.7%)	5 (71.4%)	7 (70%)	0.998
	Third trimester	0 (0%)	2 (28.6%)	2 (20%)	0.998
Age	μ ± SD (IQR)	38.49 ± 13.22 (28–49)	24.11 ± 4.32 (21–26)	29.08 ± 10.93 (22–31)	**<0.001**
State	Hessen	307 (95.3%)	570 (93.8%)	877 (94.3%)	*Reference*
	Nordrhein Westfalen	4 (1.2%)	13 (2.1%)	17 (1.8%)	0.331
	Rheinland-Pfalz	2 (0.6%)	8 (1.3%)	10 (1.1%)	0.334
	Bayern	2 (0.6%)	6 (1%)	8 (0.9%)	0.558
	Baden Württemberg	1 (0.3%)	5 (0.8%)	6 (0.6%)	0.367
	Niedersachsen	1 (0.3%)	3 (0.5%)	4 (0.4%)	0.678
	Brandenburg	0 (0%)	2 (0.3%)	2 (0.2%)	0.988
	Mecklenburg-Vorpommern	2 (0.6%)	0 (0%)	2 (0.2%)	0.987
	Schleswig-Holstein	2 (0.6%)	0 (0%)	2 (0.2%)	0.987
	Berlin	0 (0%)	1 (0.2%)	1 (0.1%)	0.992
	Saarland	1 (0.3%)	0 (0%)	1 (0.1%)	0.991

*Chi-squared test (χ^2^), Mann-Whitney test (U), and logistic regression were used with a significance level (Sig.) ≤ 0.05.*

† *Female participants.*

‡ *Pregnant participants. The bold values indicate the significant values.*

The * symbol indicates Fisher's exact test.

### COVID-19-Related Anamnesis

Only 55 (5.9%) participants had been previously infected by SARS-CoV-2, of which the vast majority were infected before the first dose (90.9%), followed by those who were infected after the second dose (7.3%) and between the first and second doses (1.8%). According to the Australian guidelines for the clinical care of people with COVID-19, more than half of the infected participants (50.9%) had mild infection, 43.6% had moderate infection, 3.6% were asymptomatic, and 1.8% had severe infection (31). The most common clinical feature was headache (62.3%), followed by fatigue (60.4%), cough (58.5%), loss of taste/smell (54.7%), sore throat (49.1%), myalgia (49.1%), and fever/chills (45.3%). There was no statistically significant difference found between employees and students in terms of infection rate, onset or clinical severity (Table 2).

**Table 2:** COVID-19-related anamnesis of German university students and employees participating in the COVID-19 Vaccine Booster Hesitancy (VBH) Survey, december 2021 (*n* = 930).

**Variable**	**Outcome**	**Employees** **(*n* = 322)**	**Students** **(*n* = 608)**	**Total** **(*n* = 930)**	***Sig*.**
Infection	No	308 (95.7%)	567 (93.3%)	875 (94.1%)	0.141
	Yes^†^	14 (4.3%)	41 (6.7%)	55 (5.9%)	
^†^Onset	Before first dose	12 (85.7%)	38 (92.7%)	50 (90.9%)	*Reference*
	Between 1^st^ and 2^nd^ Dose	1 (7.1%)	0 (0%)	1 (1.8%)	0.991
	After second dose	1 (7.1%)	3 (7.3%)	4 (7.3%)	0.964
^†^Severity	Asymptomatic	0 (0%)	2 (4.9%)	2 (3.6%)	*Reference*
	Mild	7 (50%)	21 (51.2%)	28 (50.9%)	0.995
	Moderate	6 (42.9%)	18 (43.9%)	24 (43.6%)	0.995
	Severe	1 (7.1%)	0 (0%)	1 (1.8%)	0.994
^†^Symptoms	Fever / Chills	7 (50%)	17 (43.6%)	24 (45.3%)	0.679
	Cough	10 (71.4%)	21 (53.8%)	31 (58.5%)	0.252
	Dyspnoea	3 (21.4%)	17 (43.6%)	20 (37.7%)	0.142
	Fatigue	8 (57.1%)	24 (61.5%)	32 (60.4%)	0.773
	Myalgia	7 (50%)	19 (48.7%)	26 (49.1%)	0.934
	Headache	8 (57.1%)	25 (64.1%)	33 (62.3%)	0.645
	Loss of taste / smell	6 (42.9%)	23 (59%)	29 (54.7%)	0.299
	Sore throat	5 (35.7%)	21 (53.8%)	26 (49.1%)	0.244
	Congestion	5 (35.7%)	17 (43.6%)	22 (41.5%)	0.608
	Nausea / vomiting	2 (14.3%)	3 (7.7%)	5 (9.4%)	0.599^*^
	Diarrhea	5 (35.7%)	4 (10.3%)	9 (17%)	0.044^*^
Vaccinated	Yes^‡^	314 (97.5%)	576 (94.7%)	890 (95.7%)	**0.047**
	No	8 (2.5%)	32 (5.3%)	40 (4.3%)	
^‡^Number of doses	One dose	13 (4.1%)	32 (5.6%)	45 (5.1%)	*Reference*
	Two doses	191 (60.8%)	412 (71.5%)	603 (67.8%)	0.698
	Three doses	110 (35%)	132 (22.9%)	242 (27.2%)	0.042
^‡^First dose	BNT162b2	209 (66.6%)	415 (72%)	624 (70.1%)	*Reference*
	mRNA-1273	34 (10.8%)	65 (11.3%)	99 (11.1%)	0.868
	AZD1222	59 (18.8%)	66 (11.5%)	125 (14%)	0.004
	Ad26.COV2.S	12 (3.8%)	30 (5.2%)	42 (4.7%)	0.513
^‡^Second dose	BNT162b2	249 (82.7%)	463 (85.1%)	712 (84.3%)	*Reference*
	mRNA-1273	39 (13%)	70 (12.9%)	109 (12.9%)	0.869
	AZD1222	13 (4.3%)	11 (2%)	24 (2.8%)	0.059
^‡^Third dose	BNT162b2	89 (80.9%)	119 (90.2%)	208 (86%)	**0.039**
	mRNA-1273	21 (19.1%)	13 (9.8%)	34 (14%)	
^‡^Hospitalization	Yes	2 (0.6%)	3 (0.5%)	5 (0.6%)	1.000^*^
^‡^Medical care	Yes	55 (17.5%)	127 (22%)	182 (20.4%)	0.109

Chi-squared test (χ^2^), Fisher's exact test (*) and logistic regression were used with a significance level (Sig.) ≤ 0.05.

† *Participants who were infected with COVID-19.*

‡ *Participants who received COVID-19 vaccine. The bold values indicate the significant values*.

Regarding the COVID-19 vaccine-related anamnesis, the vast majority of the participants (95.7%) reported receiving at least one dose of COVID-19 vaccines. Over two-thirds (67.8%) received two doses, 27.2% received three doses, and only 5.1% received one dose. The students had a significantly (*Sig*. = 0.047) lower vaccination rate (94.7%) than the employees (97.5%). Similarly, the students had a significantly (*Sig*. = 0.042) lower level of third dose uptake (22.9%) than the employees (35%). The BNT162b2 vaccine was the most common vaccine received for the first dose (70.1%), the second dose (84.3%), and the third dose (86%). The number of participants who received AZD1222 had dramatically dropped from the first dose (*n* = 125) to the second dose (*n* = 24). Ad26.COV2.S was exclusively administered for the first dose. While only 5 (0.6%) participants reported having post-vaccination side effects that required hospital admission, 182 (20.4%) reported seeking medical care/treatment to manage the post-vaccination side effects (Table 2).

### COVID-19 Vaccine Booster Hesitancy

Most participants indicated their acceptance to receive COVID-19 VB (87.8%), while 7.7% rejected it, and 4.4% were still hesitant. The students were significantly (*Sig*. < 0.001) more inclined to reject (9.9%) compared to the employees (3.7%), and they were also less likely to accept (86.3%) than the employees (90.7%). When asked about their reasons to accept COVID-19 VB, the most commonly cited promoter was protection of one's own health (95.6%), followed by community health protection (91.6%), family health protection (91.2%), and easier social life (57.9%). The students had significantly higher likelihood of selecting “avoid testing” and “easier social life” (24.8 and 63.8%, respectively) as promoters for COVID-19 VB compared to the employees (18.5 and 47.3%, respectively). The employer's endorsement was reported by only 3.7% of those who accepted to receive COVID-19 VB. Avoidance of economic collapse, returning to normal life, and protecting the elderly and the vulnerable individuals were added by participants as additional comments (Table 3).

**Table 3:** COVID-19 Vaccine-related attitudes of German university students and employees participating in the COVID-19 Vaccine Booster Hesitancy (VBH) Survey, december 2021 (*n* = 930).

**Variable**	**Outcome**	**Employees** **(*n* = 322)**	**Students** **(*n* = 608)**	**Total** **(*n* = 930)**	***Sig*.**
Attitudes	Rejection	12 (3.7%)	60 (9.9%)	72 (7.7%)	**<0.001**
	Hesitancy	18 (5.6%)	23 (3.8%)	41 (4.4%)	0.202
	Acceptance^†^	292 (90.7%)	525 (86.3%)	817 (87.8%)	**0.054**
^†^Promoter	Self-protection	282 (96.6%)	499 (95%)	781 (95.6%)	0.308
	Patient / Client safety	119 (40.8%)	174 (33.1%)	293 (35.9%)	**0.030**
	Family safety	270 (92.5%)	475 (90.5%)	745 (91.2%)	0.336
	Community safety	263 (90.1%)	485 (92.4%)	748 (91.6%)	0.255
	Avoid testing	54 (18.5%)	130 (24.8%)	184 (22.5%)	**0.040**
	Easier social life	138 (47.3%)	335 (63.8%)	473 (57.9%)	**<0.001**
	Employer	15 (5.1%)	15 (2.9%)	30 (3.7%)	0.097

*Chi-squared test (χ^2^) was used with a significance level (Sig.) ≤ 0.05. The bold values indicate the significant values. The meaning of the symbol ^†^ provided are Vaccine-accepting group*.

On evaluating perceived effectiveness, the vast majority (90.1%) of the participants agreed that COVID-19 VB were capable of preventing severe illness, while only 63.4% and 60.3% agreed that COVID-19 VB were capable of preventing symptomatic infection and community transmission, respectively. As low as 7.6% of the participants indicated that they would not take COVID-19 VB until they found reliable evidence confirming their capacity of controlling the emerging variants/mutations. Regarding the perceived safety, most participants (89.1%) agreed that the currently available COVID-19 VB were as safe as the primer doses, and 70.2% disagreed that COVID-19 VB would cause more severe post-vaccination side effects compared to the primer doses. While 85.2% of the participants believed that the benefits of COVID-19 VB outweighed their risks, only 64.3% agreed with the notion that they should be prioritized to receive COVID-19 VB. More than one third of the participants (36.2%) disagreed to receive COVID-19 VB due to ethical dilemma of global vaccine justice. Similarly, 35.7% disagreed to receive COVID-19 VB due to ethical dilemma of national vaccine justice. Only 15.8% of the participants agreed with the notion that they should receive a different vaccine type as VB, and only 15.1% thought the government should purchase a certain vaccine type for COVID-19 VB. The most frequently suggested vaccine was BNT16b2 (73.7%) followed by mRNA-1273 (23.5%), AZD1222 (1.6%) and Ad26.COV2.S (1.2%) (Table 4).

**Table 4:** Psychosocial drivers of German university students and employees participating in the COVID-19 Vaccine Booster Hesitancy (VBH) Survey, december 2021 (*n* = 930).

**Variable**	**Outcome**	**Frequency (*n*)**	**Percentage (*%*)**
**[Severe illness]** I think that the currently available booster doses (third shots) can protect me from severe COVID-19 infection.	Disagreement	40	4.3%
	Not sure	52	5.6%
	Agreement	838	90.1%
**[Symptomatic infection]** I think that the currently available booster doses (third shots) can protect me from symptomatic COVID-19 infection.	Disagreement	98	10.5%
	Not sure	242	26%
	Agreement	590	63.4%
**[Community transmission]** I think that the currently available booster doses (third shots) can prevent community transmission of SARS-CoV-2 and its variants.	Disagreement	150	16.1%
	Not sure	219	23.5%
	Agreement	561	60.3%
**[Mutations control]** I will not take the third shoot (booster dose) until I find reliable evidence confirming their ability to tackle the new circulating variants of SARS-CoV-2.	Disagreement	771	82.9%
	Not sure	88	9.5%
	Agreement	71	7.6%
**[Equal safety]** I think that the currently available booster doses (third shots) are as safe as the previous doses of COVID-19 vaccines.	Disagreement	38	4.1%
	Not sure	63	6.8%
	Agreement	829	89.1%
**[Non-inferior safety]** I think that the currently available booster doses (third shots) will cause more severe side effects compared to the previous doses and that will interfere with my daily routine.	Disagreement	653	70.2%
	Not sure	184	19.8%
	Agreement	93	10%
**[Risk-benefit ratio]** I believe that the benefits of booster doses (third shots) outweigh their risks.	Disagreement	73	7.8%
	Not sure	65	7%
	Agreement	792	85.2%
**[Self-prioritization]** I agree to be prioritized to receive the currently available booster doses (third shorts).	Disagreement	162	17.4%
	Not sure	170	18.3%
	Agreement	598	64.3%
**[Global vaccine justice]** I agree to receive the booster dose (third shot) of COVID-19 vaccine even after learning that administering third shots in developed economies may deprive masses in the third world from getting even the first dose.	Disagreement	337	36.2%
	Not sure	328	35.3%
	Agreement	265	28.5%
**[National vaccine Justice]** I agree to receive the booster dose (third shot) of COVID-19 vaccine even after learning that this may affect the accessibility of some population groups to the vaccine.	Disagreement	332	35.7%
	Not sure	346	37.2%
	Agreement	252	27.1%
**[Vaccine satisfaction]** I think that I should receive a different vaccine type / brand for the booster dose from the previous doses.	Disagreement	446	48%
	Not sure	337	36.2%
	Agreement	147	15.8%
**[Vaccine selectivity]** I think that the government should purchase a certain vaccine type / brand for the booster doses.	Disagreement	439	47.2%
	Not sure	351	37.7%
	Agreement	140	15.1%
^†^**[Preferred vaccine]** Which vaccine type should be promoted for booster doses?	BNT162b2	182	73.7%
	mRNA-1273	58	23.5%
	AZD1222	4	1.6%
	Ad26.COV2.S	3	1.2%

*The meaning of the symbol ^†^ provided are who wanted to take certain vaccine*.

When comparing the psychosocial drivers across gender, males had significantly higher levels of agreement with the notions that COVID-19 VB were effective against symptomatic infection (*Sig*. = 0.001), they would not receive COVID-19 VB until its effectiveness against mutations is confirmed (*Sig*. = 0.031), and they would prefer to receive different vaccine type for COVID-19 VB (*Sig*. < 0.001). The differences between employees and students were statistically significant in terms of self-prioritization (*Sig*. < 0.001), as 72% of employees *vs*. 60.2% of students agreed that they should be prioritized to receive COVID-19 VB. Additionally, students were significantly more inclined to reject receiving COVID-19 VB due to global vaccine justice dilemma (*Sig*. < 0.001) and national vaccine justice dilemma (*Sig*. < 0.001). All the surveyed psychosocial drivers were significantly different between the participants who received primer doses only and those who received booster doses, except for two drivers: vaccine satisfaction and vaccine selectivity (Table 5).

**Table 5:** Psychosocial drivers of German university students and employees participating in the COVID-19 Vaccine Booster Hesitancy (VBH) Survey stratified by gender, employment status, and number of doses, december 2021 (*n* = 930).

**Variable**	**Outcome**	**Females** **(*n* = 682)**	**Males** **(*n* = 232)**	***Sig*.**	**Employees** **(*n* = 322)**	**Students (*n* = 608)**	***Sig*.**	**Primer** **(*n* = 621)**	**Booster** **(*n* = 242)**	***Sig*.**
Severe illness	Disagreement	29 (4.3%)	8 (3.4%)	0.591	8 (2.5%)	32 (5.3%)	**0.047**	20 (3.2%)	0 (0%)	**0.005**
	Not sure	39 (5.7%)	12 (5.2%)	0.754	27 (8.4%)	25 (4.1%)	0.010	30 (4.8%)	7 (2.9%)	0.207
	Agreement	614 (90%)	212 (91.4%)	0.547	287 (89.1%)	551 (90.6%)	0.468	571 (91.9%)	235 (97.1%)	**0.006**
Symptomatic infection	Disagreement	78 (11.4%)	18 (7.8%)	0.114	28 (8.7%)	70 (11.5%)	0.183	61 (9.8%)	8 (3.3%)	**0.002**
	Not sure	190 (27.9%)	46 (19.8%)	**0.016**	91 (28.3%)	151 (24.8%)	0.257	164 (26.4%)	61 (25.2%)	0.718
	Agreement	414 (60.7%)	168 (72.4%)	**0.001**	203 (63%)	387 (63.7%)	0.855	396 (63.8%)	173 (71.5%)	**0.032**
Community transmission	Disagreement	105 (15.4%)	41 (17.7%)	0.414	44 (13.7%)	106 (17.4%)	0.137	93 (15%)	17 (7%)	**0.002**
	Not sure	172 (25.2%)	43 (18.5%)	**0.038**	77 (23.9%)	142 (23.4%)	0.849	152 (24.5%)	55 (22.7%)	0.589
	Agreement	405 (59.4%)	148 (63.8%)	0.235	201 (62.4%)	360 (59.2%)	0.341	376 (60.5%)	170 (70.2%)	**0.008**
Mutations control	Disagreement	567 (83.1%)	193 (83.2%)	0.985	276 (85.7%)	495 (81.4%)	0.098	509 (82%)	228 (94.2%)	**<0.001**
	Not sure	71 (10.4%)	14 (6%)	**0.047**	27 (8.4%)	61 (10%)	0.414	66 (10.6%)	7 (2.9%)	**<0.001**
	Agreement	44 (6.5%)	25 (10.8%)	**0.031**	19 (5.9%)	52 (8.6%)	0.147	46 (7.4%)	7 (2.9%)	**0.013**
Equal safety	Disagreement	26 (3.8%)	8 (3.4%)	0.800	9 (2.8%)	29 (4.8%)	0.148	16 (2.6%)	1 (0.4%)	0.052^*^
	Not sure	46 (6.7%)	15 (6.5%)	0.883	27 (8.4%)	36 (5.9%)	0.155	46 (7.4%)	6 (2.5%)	**0.006**
	Agreement	610 (89.4%)	209 (90.1%)	0.781	286 (88.8%)	543 (89.3%)	0.820	559 (90%)	235 (97.1%)	**<0.001**
Non-inferior safety	Disagreement	492 (72.1%)	156 (67.2%)	0.156	218 (67.7%)	435 (71.5%)	0.223	423 (68.1%)	201 (83.1%)	**<0.001**
	Not sure	125 (18.3%)	52 (22.4%)	0.174	71 (22%)	113 (18.6%)	0.207	146 (23.5%)	22 (9.1%)	**<0.001**
	Agreement	65 (9.5%)	24 (10.3%)	0.718	33 (10.2%)	60 (9.9%)	0.854	52 (8.4%)	19 (7.9%)	0.802
Risk-benefit ratio	Disagreement	54 (7.9%)	16 (6.9%)	0.613	20 (6.2%)	53 (8.7%)	0.176	33 (5.3%)	3 (1.2%)	**0.007**
	Not sure	48 (7%)	15 (6.5%)	0.766	24 (7.5%)	41 (6.7%)	0.686	43 (6.9%)	13 (5.4%)	0.406
	Agreement	580 (85%)	201 (86.6%)	0.552	278 (86.3%)	514 (84.5%)	0.464	545 (87.8%)	226 (93.4%)	**0.016**
Self-prioritization	Disagreement	111 (16.3%)	44 (19%)	0.346	36 (11.2%)	126 (20.7%)	**<0.001**	101 (16.3%)	17 (7%)	**<0.001**
	Not sure	134 (19.6%)	34 (14.7%)	0.090	54 (16.8%)	116 (19.1%)	0.386	126 (20.3%)	31 (12.8%)	**0.011**
	Agreement	437 (64.1%)	154 (66.4%)	0.526	232 (72%)	366 (60.2%)	**<0.001**	394 (63.4%)	194 (80.2%)	**<0.001**
Global vaccine justice	Disagreement	243 (35.6%)	85 (36.6%)	0.782	91 (28.3%)	246 (40.5%)	**<0.001**	240 (38.6%)	50 (20.7%)	**<0.001**
	Not sure	260 (38.1%)	64 (27.6%)	**0.004**	125 (38.8%)	203 (33.4%)	0.099	222 (35.7%)	94 (38.8%)	0.397
	Agreement	179 (26.2%)	83 (35.8%)	**0.006**	106 (32.9%)	159 (26.2%)	**0.030**	159 (25.6%)	98 (40.5%)	**<0.001**
National vaccine justice	Disagreement	239 (35%)	83 (35.8%)	0.840	90 (28%)	242 (39.8%)	**<0.001**	240 (38.6%)	44 (18.2%)	**<0.001**
	Not sure	266 (39%)	77 (33.2%)	0.114	136 (42.2%)	210 (34.5%)	**0.021**	226 (36.4%)	109 (45%)	**0.019**
	Agreement	177 (26%)	72 (31%)	0.133	96 (29.8%)	156 (25.7%)	0.175	155 (25%)	89 (36.8%)	**<0.001**
Vaccine satisfaction	Disagreement	350 (51.3%)	91 (39.2%)	**0.001**	126 (39.1%)	320 (52.6%)	**<0.001**	305 (49.1%)	108 (44.6%)	0.236
	Not sure	245 (35.9%)	84 (36.2%)	0.938	129 (40.1%)	208 (34.2%)	0.077	218 (35.1%)	97 (40.1%)	0.172
	Agreement	87 (12.8%)	57 (24.6%)	**<0.001**	67 (20.8%)	80 (13.2%)	**0.002**	98 (15.8%)	37 (15.3%)	0.858
Vaccine selectivity	Disagreement	311 (45.6%)	120 (51.7%)	0.107	154 (47.8%)	285 (46.9%)	0.782	281 (45.2%)	123 (50.8%)	0.140
	Not sure	259 (38%)	85 (36.6%)	0.716	128 (39.8%)	223 (36.7%)	0.358	248 (39.9%)	82 (33.9%)	0.100
	Agreement	112 (16.4%)	27 (11.6%)	0.080	40 (12.4%)	100 (16.4%)	0.102	92 (14.8%)	37 (15.3%)	0.861

Chi-squared test (χ^2^) and Fisher's exact test (*) were used with a significance level (Sig.) ≤ 0.05. The bold values indicate the significant values.

### Determinants of COVID-19 VBH

The differences between females and males were not statistically significant in terms of COVID-19 VB attitudes, even though the LGBTQ+ participants were more likely to reject COVID-19 VB (25%) when compared to females (7.2%) and males (8.2%). The pregnant participants (30%) were significantly (*Sig*. = 0.029) more inclined to reject COVID-19 VB compared to the non-pregnant participants (6.8%). The participants who had been previously infected (76.4%) were significantly (*Sig*. = 0.007) less inclined to accept COVID-19 VB than those who had not been infected (88.6%). There were no statistically significant differences across onset or clinical severity groups in terms of COVID-19 VB attitudes (Table 6).

**Table 6:** Determinants of COVID-19 vaccine-related attitudes of German university students and employees participating in the COVID-19 Vaccine Booster Hesitancy (VBH) Survey, december 2021 (*n* = 930).

	**Variable**	**Outcome**	**Rejection** **(*n* = 72)**	***Sig*.**	**Hesitancy** **(*n* = 41)**	***Sig*.**	**Acceptance** **(*n* = 817)**	***Sig*.**
Demographic determinants	Gender	Female^†^	49 (7.2%)	*Reference*	31 (4.5%)	*Reference*	602 (88.3%)	*Reference*
		Male	19 (8.2%)	0.615	10 (4.3%)	0.881	203 (87.5%)	0.755
		LGBTQ+	4 (25%)	**0.014**	0 (0%)	0.988	12 (75%)	0.119
	^†^Pregnancy	No	46 (6.8%)	0.029^*^	30 (4.5%)	0.374^*^	596 (88.7%)	0.021^*^
		Yes^‡^	3 (30%)		1 (10%)		6 (60%)	
	^‡^Trimester	First trimester	0 (0%)	*Reference*	1 (100%)	*Reference*	0 (0%)	*Reference*
		Second trimester	3 (42.9%)	0.998	0 (0%)	1.000	4 (57.1%)	0.998
		Third trimester	0 (0%)	1.000	0 (0%)	1.000	2 (100%)	0.996
COVID-19-related anamnesis	Infection	No	66 (7.5%)	0.429^*^	34 (3.9%)	0.008^*^	775 (88.6%)	**0.007**
		Yes ^ψ^	6 (10.9%)		7 (12.7%)		42 (76.4%)	
	^ψ^nset	Before first dose	6 (12%)	*Reference*	6 (12%)	*Reference*	38 (76%)	*Reference*
		Between 1^st^ & 2^nd^ Dose	0 (0%)	0.998	0 (0%)	0.995	1 (100%)	0.992
		After second dose	0 (0%)	0.996	1 (25%)	0.469	3 (75%)	0.964
	^ψ^Severity	Asymptomatic	1 (50%)	*Reference*	0 (0%)	*Reference*	1 (50%)	*Reference*
		Mild	4 (14.3%)	0.237	2 (7.1%)	0.996	22 (78.6%)	0.382
		Moderate	1 (4.2%)	0.072	4 (16.7%)	0.995	19 (79.2%)	0.374
		Severe	0 (0%)	0.994	1 (100%)	0.994	0 (0%)	0.994
	^ψ^Symptoms	Fever / Chills	0 (0%)	0.056^*^	4 (16.7%)	0.688^*^	20 (83.3%)	0.344
		Cough	2 (6.5%)	0.638^*^	6 (19.4%)	0.218^*^	23 (74.2%)	0.740^*^
		Dyspnoea	1 (5%)	0.639^*^	2 (10%)	0.697^*^	17 (85%)	0.500^*^
		Fatigue	1 (3.1%)	0.074^*^	5 (15.6%)	0.690^*^	26 (81.3%)	0.507^*^
		Myalgia	2 (7.7%)	1.000^*^	4 (15.4%)	0.704^*^	20 (76.9%)	0.941
		Headache	2 (6.1%)	0.354^*^	5 (15.2%)	0.697^*^	26 (78.8%)	0.748^*^
		Loss of taste / Smell	2 (6.9%)	0.649^*^	3 (10.3%)	0.688^*^	24 (82.8%)	0.302
		Sore throat	3 (11.5%)	0.669^*^	5 (19.2%)	0.250^*^	18 (69.2%)	0.165
		Congestion	1 (4.5%)	0.389^*^	5 (22.7%)	0.113^*^	16 (72.7%)	0.524^*^
		Nausea / Vomiting	0 (0%)	1.000^*^	3 (60%)	0.013^*^	2 (40%)	0.070^*^
		Diarrhea	1 (11.1%)	1.000^*^	4 (44.4%)	0.012^*^	4 (44.4%)	0.020^*^
Vaccine anamnesis	Vaccinated	No	39 (97.5%)	<0.001^*^	0 (0%)	0.252^*^	1 (2.5%)	**<** 0.001^*^
		Yes	33 (3.7%)		41 (4.6%)		816 (91.7%)	
	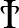 Number of doses	One dose	9 (20%)	*Reference*	8 (17.8%)	*Reference*	28 (62.2%)	*Reference*
		Two doses	22 (3.6%)	**<0.001**	31 (5.1%)	**0.001**	550 (91.2%)	**<0.001**
		Three doses	2 (0.8%)	**<0.001**	2 (0.8%)	**<0.001**	238 (98.3%)	**<0.001**
	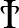 Hospitalization	No	33 (3.7%)	1.000^*^	38 (4.3%)	<0.001^*^	814 (92%)	0.005^*^
		Yes	0 (0%)		3 (60%)		2 (40%)	
	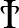 Medical care	No	21 (3%)	**0.021**	29 (4.1%)	0.152	658 (92.9%)	**0.008**
		Yes	12 (6.6%)		12 (6.6%)		158 (86.8%)	

Chi-squared test (χ^2^), Fisher's exact test (*) and logistic regression were used with a significance level (Sig.) ≤ 0.05.

† *Female participants.*

‡ *Pregnant participants.*

Ψ *Participants who were infected with COVID-19.*

*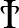 Participants who received COVID-19 vaccine. The bold values indicate the significant values*.

The participants who had been previously vaccinated (91.7%) were significantly (*Sig*. < 0.001) more inclined to accept COVID-19 VB than those who had not been vaccinated (2.5%). Receiving one dose was significantly associated with decreased likelihood of COVID-19 VB acceptance (62.2%) compared to receiving two doses (91.2%; *Sig*. < 0.001) and three doses (98.3%; *Sig*. < 0.001). Suffering from severe post-vaccination side effects that required hospital admission was significantly (*Sig*. = 0.005) associated with decreased likelihood of COVID-19 VB acceptance (40%) compared to having no history of hospitalization (92%). Similarly, seeking medical care/treatment to manage post-vaccination side effects was significantly (*Sig*. = 0.008) associated with decreased likelihood of COVID-19 VB acceptance (86.8%) compared to having no history of severe side effects (92.9%) (Table 6).

### Regression Analysis of COVID-19 VBH

The binary logistic regression confirmed the role of previous COVID-19 infection, receiving only one dose, hospital admission, and seeking medical care/treatment in decreasing the odds of accepting COVID-19 VB; therefore, these four variables were controlled while calculating the adjusted odds ratio (AOR) of the psychosocial drivers (Table 7).

**Table 7:** Regression analysis of COVID-19 vaccine-related acceptance demographic and anamnestic drivers of German university students and employees participating in the COVID-19 Vaccine Booster Hesitancy (VBH) Survey, december 2021 (*n* = 930).

**Predictor**	**B (SE)**	**Wald**	**OR (CI 95%)**	***Sig*.**
Male (*vs*. Female)	−0.07 (0.23)	0.10	0.93 (0.59–1.46)	0.755
Age group: >29 yo (≤ 29 yo)	0.07 (0.23)	0.10	1.07 (0.69–1.67)	0.749
Status: employee (*vs*. student)	0.43 (0.23)	3.66	1.54 (0.99–2.39)	0.056
Infection: no (*vs*. yes)	0.88 (0.34)	6.83	2.40 (1.25–4.62)	**0.009**
Number of doses: two (*vs*. one)	1.84 (0.34)	29.4	6.3 (3.24–12.26)	**<0.001**
Number of doses: three (*vs*. one)	3.59 (0.59)	36.89	36.13 (11.35–114.94)	**<0.001**
Hospital: no (*vs*. yes)	2.85 (0.92)	9.54	17.2 (2.83–104.62)	**0.002**
Care: no (*vs*. yes)	0.69 (0.26)	6.9	2 (1.19–3.35)	**0.009**

*Binary logistic regression had been used with a significance level (Sig.) ≤ 0.05. The bold values indicate the significant values*.

Agreement with the notion that COVID-19 VB were capable of preventing severe illness had AOR of 47.65 (CI 95%: 23.65–96.49) for COVID-19 VB acceptance. Similarly, agreement with the notion that COVID-19 VB were capable of preventing symptomatic infection and community transmission had AORs of 9.87 (CI 95%: 5.20–18.71) and 5.34 (CI 95%: 3.00–9.49) for COVID-19 VB acceptance, respectively. The participants who disagreed to receive COVID-19 VB until its effectiveness against variants is confirmed had AOR of 19.12 (CI 95%: 10.57–34.55) for COVID-19 VB acceptance. Agreement with equal safety and disagreement with severe side effects of COVID-19 VB had AORs of 24.27 (CI 95%: 12.93–45.56) and 6.68 (CI 95%: 3.81–11.71) for COVID-19 VB acceptance, respectivel (Table 8).

**Table 8:** Regression analysis of COVID-19 vaccine-related acceptance psychosocial drivers of German university students and employees participating in the COVID-19 Vaccine Booster Hesitancy (VBH) Survey, december 2021 (*n* = 930).

**Predictor**	**B (SE)**	**Wald**	**AOR (CI 95%)**	***Sig*.**
Severe illness: agree	3.86 (0.36)	115.25	47.65 (23.65–96.49)	**<0.001**
Symptomatic infection: agree	2.29 (0.33)	49.17	9.87 (5.20–18.71)	**<0.001**
Community transmission: agree	1.68 (0.29)	32.56	5.34 (3.00–9.49)	**<0.001**
Mutations control: disagree	2.95 (0.30)	95.41	19.12 (10.57–34.55)	**<0.001**
Equal safety: agree	3.19 (0.32)	98.53	24.27 (12.93–45.56)	**<0.001**
Non-inferior safety: disagree	1.90 (0.29)	44.05	6.68 (3.81–11.71)	**<0.001**
Risk-benefit ratio: agree	4.65 (0.39)	143.04	104.55 (48.80–224.01)	**<0.001**
Self-prioritization: agree	2.74 (0.38)	53.13	15.43 (7.39–32.21)	**<0.001**
Global vaccine justice: agree	1.89 (0.53)	12.95	6.65 (2.37–18.65)	**<0.001**
National vaccine justice: agree	2.16 (0.60)	12.91	8.65 (2.67–28.07)	**<0.001**
Vaccine satisfaction: disagree	0.11 (0.26)	0.17	1.11 (0.67–1.86)	0.680
Vaccine selectivity: disagree	−0.06 (0.26)	0.05	0.95 (0.57–1.57)	0.831

*Binary logistic regression had been used with a significance level (Sig.) ≤ 0.05 and adjusted for infection, number of doses, hospitalization, and medical care. The bold values indicate the significant values*.

The highest AOR for COVID-19 VB acceptance was found in case of agreement with the risk-benefit ratio notion which was 104.55 (CI 95%: 48.80–224.01). Agreement with the self-prioritization notion had also AOR of 15.43 (CI 95%: 7.39–32.21) for COVID-19 VB acceptance. Ignoring the ethical dilemmas of vaccine justice was indicated by agreement of receiving the vaccine despite knowing that it might affect access of other populations globally or other population groups nationally to primer doses of COVID-19 vaccines. Hence, ignoring the ethical dilemmas of vaccine justice globally and nationally had AORs of 6.65 (CI 95%: 2.37–18.65) and 8.65 (CI 95%: 2.67–28.07) for COVID-19 VB acceptance, respectively. Vaccine satisfaction and vaccine selectivity did not have significant impact on COVID-19 VB acceptance (Table 8).

## Discussion

The overall COVID-19 VB acceptance among German university students and employees was satisfactory (87.8%) and induced by various altruistic promoters, e.g., family health protection, community health protection, and patients' health protection. Compared to our findings, Rzymski et al. (32, 33) and Klugar et al. (15) revealed lower COVID-19 VB acceptance levels among Polish (71%) and Czech (71.3%) populations in September 2021 and November 2021, respectively (15, 32). Additionally, Alhasan et al. (34) found that only 55.3% of the Saudi healthcare workers indicated their acceptance to receive COVID-19 VB when they were surveyed in August 2021 (34). Similarly, Yadete et al. (35) found that about 62% of a representative sample of the American adult population accepted COVID-19 VB in July 2021 (35). On the other hand, 84.8% of the Chinese adults were willing to receive COVID-19 VB in June 2021, and 88.9% of the American medical students were in favor of receiving COVID-19 VB in Spring 2021 (36, 37).

By December 20^th^, 2021, there had been 6,991,381 confirmed COVID-19 cases in Germany representing 8.3% of the total German population, which is larger than the proportion of the previously infected participants (5.9%) in our sample (38). This difference can be attributed to the age structure of our sample which was younger than the age structure of confirmed cases in the general population, with bearing in mind that younger age groups are associated with asymptomatic and mild infections which tend to be undetected/missed (39). According to RKI, the largest age group of confirmed COVID-19 cases in Germany is 35–59 years, while the mean age of our participants was 29.08 ± 10.93 years with an interquartile range of 22–31 years (40).

In our sample, the most administered COVID-19 vaccine type was BNT162b2 (78.1%), followed by mRNA-1273 (12.2%), AZD1222 (7.5%), and Ad26.COV2.S (2.1%). The same order was found among the general German population; by December 26^th^, 2021, there had been 164.3 million doses of COVID-19 vaccines in Germany with BNT162b2 being the most commonly administered type (72.1%), followed by mRNA-1273 (15.9%), AZD1222 (8.8%), and Ad26.COV2.S (3.2%) (41). While the crude vaccination rate, i.e., receiving at least one vaccine shot, in Germany was 74.1%, the crude vaccination rate in our sample was 95.7%, thus suggesting that our sample may represent an above-average subset of the German population in terms of vaccine acceptance that could be attributed to their supposedly high level of health literacy (41).

One of the public health challenges that happened amid 2021 and portrayed by our findings is the dramatic decline of public demand for AZD1222 vaccine (commonly known as AstraZeneca-Oxford COVID-19 vaccine), which had been received by 125 participants for the first dose and only 24 participants for the second dose with 80.8% of decline. The “AstraZeneca catastrophe” had been triggered by the initial decision of the German Standing Committee on Vaccination (STIKO) to deliver AZD1222 for those below 65 years of age due to incomplete data of pre-authorization trials (42). Few weeks later, the situation was further complicated by the emergence of few thrombotic events among the individuals who received AZD1222 that made the European Medicines Agency (EMA) recommends temporary suspension of AZD1222 mass administration until those reports were reviewed (43). However, the EMA's safety committee recommended the continuation of AZD1222 use for mass inoculation as its benefits were found to weigh its risks, the public demand continued to fall in western Europe including Germany to the point that the Federal Government of Germany donated all remaining AZD1222 doses to COVAX consortium in August 2021 (44).

Living with an immunocompromised family member was significantly associated with increased likelihood of COVID-19 VB acceptance among American adults (35). El-Mohandes et al. (45) concluded that protection of one's own health, protection of family health, and help ending the pandemic were the major reasons for accepting COVID-19 vaccines among American adults (45). Another study of social media users in the US revealed that having a large family was associated with higher odds of COVID-19 vaccine acceptance; however, vaccine hesitancy remained existent in large families that did not have confidence in pharmaceutical industry (46). Similarly, our study revealed that protection of one's own health was the most commonly cited promoter for COVID-19 VB acceptance (95.6%), followed by protection of community health (91.6%) and protection of family health (91.2%). Moreover, our findings are in complete agreement with the results of Schernhammer et al. (47) who found that protection of one's own health and protection of family health were the most common reasons for Austrian adults to accept COVID-19 vaccines (47). Given that evidence-based communication strategies are crucial for the fight against COVID-19 vaccine hesitancy, our results underline the importance of positive emotions in enhancing vaccine uptake by highlighting the role of indirect immunity in protecting family members and beloved ones (15, 48).

COVID-19 vaccine hesitancy had decreased significantly during the lockdown period among Italian residents; therefore, Caserotti et al. (49) proposed that lockdown and the anti-pandemic restrictions can be contextual factors that induce vaccine acceptance (49). On the other hand, Fernandes et al. (50) could not find any correlation between the number of days in lockdown and vaccination intention, thus suggesting that anti-pandemic restrictions could have caused a sense of protection due to the associated decline of cases during lockdown, which may have made the people believe that vaccination was no longer necessary (50). Having easier social life with less restrictions on mobility and gatherings was one of the key promoters for COVID-19 VB acceptance (57.9%) in our sample, and avoidance of frequent testing (22.5%) was also an important promoter. These findings suggest that vaccine messaging may benefit from highlighting the social benefits of mass vaccination that can help reduce anti-pandemic restrictions dramatically.

In April 2021, the WHO issued a policy paper discussing the ethical concerns of COVID-19 mandatory vaccination and adopted no position with or against COVID-19 vaccine mandates (51). According to the WHO, vaccine mandates should not be approached as the first option for achieving public health goals, e.g., reaching herd immunity and protection of the vulnerable population, as they do compromise individual liberties and adversely affect the public trust in vaccines (51, 52). However, vaccine mandates are ideally applied when they are necessary, and when there is sufficient evidence on vaccine safety and effectiveness, private employers-imposed vaccine mandates can be counterproductive especially in western societies (51–54). The employer's endorsement was only reported by 3.7% of our participants as the promoter for COVID-19 VB acceptance, thus suggesting that vaccine mandates might not be the best option for enhancing vaccine uptake in Germany currently.

A recent cross-sectional survey from Portugal found that the perceived risk of severe COVID-19 infection was associated with increased odds of vaccine acceptance (55). This finding can be understood within the context of the 3-C model for vaccine hesitancy which implies that vaccine compliance as one of the three key elements of vaccine decision is influenced by perceived risk of disease severity (56, 57). In our sample, the belief that COVID-19 VB can prevent severe illness was a strong predictor of COVID-19 VB acceptance. Moreover, the capacity of COVID-19 VB to reduce community transmission and symptomatic infection were also strong predictors of COVID-19 VB acceptance. The growing evidence on COVID-19 vaccines effectiveness against community transmission and symptomatic infection had supported the rationale of administering booster doses for tackling two synergistically occurring phenomena; the decline of vaccine-elicited immunity and the emerging variants (33, 58–60).

Klugar et al. (15, 61) found that the perceived effectiveness of COVID-19 VB against the emerging variants of SARS-CoV-2 was not a significant predictor for COVID-19 VB acceptance among Czech healthcare workers (15). One the other hand, more than half (55.3%) of Saudi healthcare workers indicated their interest in receiving mRNA-based vaccine which is specifically developed for the Delta variant (34). In our sample, the perceived effectiveness against the emerging variants was a strong predictor for COVID-19 VB acceptance, thus underlining the importance of promoting the emerging evidence on COVID-19 VB capacity against the variants (17, 18, 62).

Safety of COVID-19 vaccines had been one of the debates that were specifically targeted by the anti-vaccination campaigns for undermining the mass vaccination efforts; therefore, the WHO called for timely and transparent dissemination of safety data of COVID-19 vaccine trials (63–66). The healthcare students who exhibited less concerns regarding COVID-19 vaccines side effects were more inclined to accept vaccination (67–69). Independent (non-sponsored) studies that are designed to monitor the self-reported side effects of COVID-19 vaccines constitute a crucial resource for active surveillance of COVID-19 vaccines safety (61, 70–76). Even minor post-vaccination side effects may cause anxiety and social burden for the recently vaccinated individuals that may hinder them from continuing their vaccination regimen; therefore, vaccine messaging should normalize the post-vaccination side effects and describe their postulated onset, duration, and severity in order to reassure the vaccinees (50, 77). The high severity of side effects following the primer doses was a key predictor for COVID-19 VB refusal among the Polish population as reported by Rzymski et al. (32). Our findings are consistent with the Polish results, as the participants who required hospital admission or sought medical care/treatment were more likely to be hesitant about receiving COVID-19 VB. These results are echoed by the finding that beliefs of equal safety and non-inferior safety were strong predictors of COVID-19 VB among our participants.

The ethical dilemma of vaccine justice has been found to be a prominent a barrier for COVID-19 VB acceptance among Czech healthcare workers as well as our sample (15). Such dilemma is triggered by the WHO stance against dissemination of booster doses in high-income countries while frontline healthcare workers in some developing countries are still queuing to receive their first dose (78–80). Dr. Ghebreyesus, the Director-General of WHO, has recently proposed a roadmap of five steps to tackle the crisis of vaccine inequity; (i) firstly, the countries which purchased enormous numbers of doses that exceed their actual populations' needs should donate those doses to the poorest countries through COVAX and the African Vaccine Acquisition Trust (AVAT), (ii) secondly, vaccine manufactures should prioritize and fulfill their contracts with COVAX and AVAT, (iii) thirdly, G7 and other vaccine-donating countries should fulfill their commitments urgently, (iv) trade barriers and export restrictions on COVID-19 vaccines should eliminated, and (v) all these recommendations need to be fulfilled simultaneously (80).

Our LGBTQ+ participants had lower levels of COVID-19 VB acceptance, which is similar to what was recently reported by Riad et al. (70, 72–74, 76, 81–83) who found that LGBTQ+ university students in the Czech Republic were less willing to receive COVID-19 vaccines (81). The recent systematic review of Garg et al. (84) on COVID-19 vaccine hesitancy among LGBTQ+ communities revealed that the common reasons for vaccine hesitancy were vaccine safety concerns, previous negative experiences with healthcare providers, and lack of inclusion of LGBTQ+ individuals in vaccine trials (84). The differences between males and females in terms of COVID-19 VB acceptance were not statistically significant among our participants. Holzmann-Littig et al. (2) found no statistically significant difference between female and male German healthcare workers in terms of COVID-19 vaccine acceptance (2). While some national and multinational studies found that males were more likely to accept COVID-19 vaccines, other studies found that females were more pro vaccination (82, 85–89).

Recent studies found that younger age was associated with lower odds of COVID-19 vaccine acceptance, which could be the main reason for why German university students had lower levels of COVID-19 VB acceptance compared to Germany university employees. Young age is associated with decreased likelihood of severe illness and disease complications, lower perceived risk of COVID-19, and delayed vaccine uptake due to the prioritization schemes of vaccine dissemination (76, 83).

### Strengths

To the best of the authors' knowledge, this study is the first to evaluate COVID-19 VBH among German population. The recruited sample reflected some key characteristics of the German population that may affect vaccination decision, e.g., infection rate, and vaccine types distribution. This study analyzed the promoters of COVID-19 VB acceptance and the psychosocial predictors that may help in tailoring vaccine messaging in Germany to boost vaccine uptake. The identity of the participants was kept anonymous in order to control Hawthorne's effect and minimize the information bias.

### Limitations

The first limitation of this study is due to its cross-sectional nature that makes it infeasible to track the changes in COVID-19 vaccination intentions longitudinally. The second limitation is due to the fact that the target population of this study, university students and employees, represent a highly educated subset of the German population with supposedly high levels of health literacy and positive vaccine intentions. Thirdly, the sample was not well-balanced across gender or pregnancy status, and the lack of information on participants' race may limit the findings generalizability. Fourthly, the vast majority of respondents were from Hessen state which may limit generalization of results at the national level.

### Implications

The results of this study call for emphasizing the expected benefits of COVID-19 vaccination in protecting one's own health, protection of family health and community health, and relieving anti-pandemic restrictions within vaccine communication strategies in Germany. Our findings recommend also that the future research on COVID-19 vaccine hesitancy should include immigrants and other minorities groups in Germany. The perceived effectiveness of COVID-19 VB against severe illness, symptomatic infection, community transmission and emerging variants needs to be highlighted in vaccine messaging. In addition, the perceived safety of COVID-19 VB and ethical dilemmas of vaccine justice need to be addressed on large scale. Mandating COVID-19 vaccines does not seem to be required in Germany, as a very tiny fraction of our participants cited employers' endorsement as a reason for COVID-19 VB acceptance.

## Conclusion

The overall COVID-19 VB acceptance among German university students and employees was satisfactory (87.8%) and induced by various altruistic promoters, e.g., family health protection, community health protection, and patients' health protection. The students (86.3%), the previously infected participants (76.4%), the participants who did not receive primer doses of COVID-19 vaccines (2.5 %), and those who were hospitalized (40%) and sought medical care/treatment after receiving primer doses (86.8%) were less likely to accept COVID-19 VB compared to the employees (90.7%), the participants who were not previously infected (88.6%) and those who received primer dose (91.7%), and the participants who were not hospitalized (92%) nor sought medical care/treatment after primer doses (92.9%), respectively. The perceived effectiveness of COVID-19 VB against severe illness, symptomatic infection, community transmission and emerging variants was a key promoter for COVID-19 VB acceptance; therefore, it needs to be highlighted in vaccine messaging. In addition, the perceived safety of COVID-19 VB and ethical dilemmas of vaccine justice need to be addressed publicly.

## Data Availability Statement

The raw data supporting the conclusions of this article will be made available by the authors, without undue reservation.

## Ethics Statement

The studies involving human participants were reviewed and approved by Ethics Committee of the Faculty of Medicine at Justus Liebig University Giessen (Ref. 259/21). The patients/participants provided their written informed consent to participate in this study.

## Author Contributions

SA and AR: conceptualization and writing—original draft preparation. KM, MK, and AR: methodology. AR: software, formal analysis, and supervision. SA and KM: validation. SA and H-PH: investigation and funding acquisition. MK and H-PH: writing—review and editing. SA: project administration. All authors have read and agreed to the published version of the manuscript.

## Funding

This work of AR was supported by Masaryk University grants number MUNI/IGA/1104/2021 and MUNI/A/1402/2021.

## Conflict of Interest

The authors declare that the research was conducted in the absence of any commercial or financial relationships that could be construed as a potential conflict of interest.

## Publisher's Note

All claims expressed in this article are solely those of the authors and do not necessarily represent those of their affiliated organizations, or those of the publisher, the editors and the reviewers. Any product that may be evaluated in this article, or claim that may be made by its manufacturer, is not guaranteed or endorsed by the publisher.
