# Supplementary material for: Prevalence and Drivers of COVID-19 Vaccine Booster Hesitancy Among German University Students and Employees
Source: Front Public Health. 2022 Apr 7;10:846861. doi: 10.3389/fpubh.2022.846861 (PMC9021373; doi:10.3389/fpubh.2022.846861)
Supplement: Supplementary file 1 [file Table_1.pdf]

Supplementary Table 1: Codebook of the dataset.

Variable Values

Value		Label
Gender	0	Female
	1	Male
	2	LGBTQ+
Pregnant	0	No
	1	Yes
Trimester	1	First Trimester
	2	Second Trimester
	3	Third Trimester
Employment	1	Employee
	2	Student
State	1	Baden Württemberg
	2	Bayern
	3	Berlin
	4	Brandenburg
	5	Hessen
	6	Mecklenburg-Vorpommern
	7	Niedersachsen
	8	Nordrhein Westfalen
	9	Rheinland-Pfalz
	10	Saarland
	11	Schleswig-Holstein
Vaccinated	0	No
	1	Yes
Doses	1	One Dose
	2	Two Doses
	3	Three Doses
Booster Vs. Primer	2	Primer
	3	Booster
One Dose	0	No
	1	Yes
Two Doses	0	No
	1	Yes
Three Doses	0	No

	1	Yes
First Dose	1	BNT162b2
	2	MRNA-1273
	3	AZD1222
	4	Ad26.COV2.S
Second Dose	1	BNT162b2
	2	MRNA-1273
	3	AZD1222
	4	Ad26.COV2.S
Third Dose	1	BNT162b2
	2	MRNA-1273
	3	AZD1222
	4	Ad26.COV2.S
Infected	0	No
	1	Yes
Infection Time	1	Before First Dose
	2	Between First and Second Dose
	3	After Second Dose
Infection Severity	0	Asymptomatic
	1	Mild
	2	Moderate
	3	Severe
Fever or Chills	0	No
	1	Yes
Cough	0	No
	1	Yes
Dyspnea	0	No
	1	Yes
Fatigue	0	No
	1	Yes
Myalgia	0	No
	1	Yes
Headache	0	No
	1	Yes
Loss of Taste / Smell	0	No
	1	Yes
Sore Throat	0	No
	1	Yes

Congestion	0	No
	1	Yes
Nausea or Vomiting	0	No
	1	Yes
Diarrhea	0	No
	1	Yes
Other Symptoms	0	No
	1	Yes
COVID-19 VB Attitudes	1	Totally Disagree
	2	Disagree
	3	Not Sure
	4	Agree
	5	Totally Agree
COVID-19 VB Position	1	Rejection
	2	Hesitancy
	3	Acceptance
Protection Own Health	0	No
	1	Yes
Protection Patient Health	0	No
	1	Yes
Protection Family Health	0	No
	1	Yes
Protection Community Health	0	No
	1	Yes
Avoidance Frequent Testing	0	No
	1	Yes
Easier Social Life	0	No
	1	Yes
Employer Endorsement	0	No
	1	Yes
Other Reasons	0	No
	1	Yes
Severe Illness	1	Disagreement
	2	Not Sure
	3	Agreement
Symptomatic Infection	1	Disagreement
	2	Not Sure
	3	Agreement

Community Transmission	1	Disagreement
	2	Not Sure
	3	Agreement
Mutations Control	1	Disagreement
	2	Not Sure
	3	Agreement
Equal Safety	1	Disagreement
	2	Not Sure
	3	Agreement
Non-inferior Safety	1	Disagreement
	2	Not Sure
	3	Agreement
Risk-benefit Ratio	1	Disagreement
	2	Not Sure
	3	Agreement
Self-prioritization	1	Disagreement
	2	Not Sure
	3	Agreement
Global Vaccine Justice	1	Disagreement
	2	Not Sure
	3	Agreement
National Vaccine Justice	1	Disagreement
	2	Not Sure
	3	Agreement
Vaccine Satisfaction	1	Disagreement
	2	Not Sure
	3	Agreement
Vaccine Selectivity	1	Disagreement
	2	Not Sure
	3	Agreement
Preferred Vaccine Type	1	BNT162b2
	2	MRNA-1273
	3	AZD1222
	4	Ad26.COV2.S
